# Sphingolipids: Key Regulators of Apoptosis and Pivotal Players in Cancer Drug Resistance

**DOI:** 10.3390/ijms15034356

**Published:** 2014-03-12

**Authors:** Paola Giussani, Cristina Tringali, Laura Riboni, Paola Viani, Bruno Venerando

**Affiliations:** Department of Medical Biotechnology and Translational Medicine, University of Milan, Segrate (Milan 20090), Italy; E-Mails: paola.giussani@unimi.it (P.G.); cristina.tringali@unimi.it (C.T.); laura.riboni@unimi.it (L.R.); paola.viani@unimi.it (P.V.)

**Keywords:** sphingolipids, ceramide, sphingosine-1P, gangliosides, globosides, sialidases

## Abstract

Drug resistance elicited by cancer cells still constitutes a huge problem that frequently impairs the efficacy of both conventional and novel molecular therapies. Chemotherapy usually acts to induce apoptosis in cancer cells; therefore, the investigation of apoptosis control and of the mechanisms used by cancer cells to evade apoptosis could be translated in an improvement of therapies. Among many tools acquired by cancer cells to this end, the de-regulated synthesis and metabolism of sphingolipids have been well documented. Sphingolipids are known to play many structural and signalling roles in cells, as they are involved in the control of growth, survival, adhesion, and motility. In particular, in order to increase survival, cancer cells: (a) counteract the accumulation of ceramide that is endowed with pro-apoptotic potential and is induced by many drugs; (b) increase the synthesis of sphingosine-1-phosphate and glucosylceramide that are pro-survivals signals; (c) modify the synthesis and the metabolism of complex glycosphingolipids, particularly increasing the levels of modified species of gangliosides such as 9-O acetylated GD3 (αNeu5Ac(2-8)αNeu5Ac(2-3)βGal(1-4)βGlc(1-1)Cer) or *N*-glycolyl GM3 (αNeu5Ac (2-3)βGal(1-4)βGlc(1-1)Cer) and de-*N*-acetyl GM3 (NeuNH(2)βGal(1-4)βGlc(1-1)Cer) endowed with anti-apoptotic roles and of globoside Gb3 related to a higher expression of the multidrug resistance gene MDR1. In light of this evidence, the employment of chemical or genetic approaches specifically targeting sphingolipid dysregulations appears a promising tool for the improvement of current chemotherapy efficacy.

## 1. Introduction

Chemotherapy is a principal approach to overcoming cancer; in particular, the employment of molecular targeted therapies has significantly improved the efficacy of chemotherapy in arresting tumour progression. Nevertheless, drug resistance remains a considerable problem because it can severely limit the effectiveness of chemotherapy.

Resistance to drugs can be intrinsic or acquired. Intrinsic resistance indicates that before receiving chemotherapy, resistance-mediating factors exist in the majority of tumour cells, which makes the therapy ineffective. By contrast, acquired drug resistance develops during treatment in tumours that are initially sensitive, and such resistance can be caused by novel mutations as well as by various other adaptive responses, such as the increased expression of therapeutic targets and the activation of alternative, compensatory signalling pathways [1]. These drug-adaptive mechanisms can explain why patients with tumour relapse usually present with more resistant disease.

A large range of molecular mechanisms are implicated in drug resistance: these include increased rates of drug efflux, alterations in drug metabolism, mutations of drug targets [1–4], the presence of cancer stem cells [5], the activation of survival signalling pathways and the inactivation of downstream death signalling pathways [4,6]. In particular, a key mechanism that leads to drug resistance is the establishment of an apoptosis resistant phenotype, because chemotherapeutic agents, despite their different mechanisms of action, generally flow into a common, final pathway of cell death: Primarily apoptosis. For these reasons, elucidating the molecular pathways that are connected to apoptosis resistance could lead to improved chemotherapy efficacy.

Among the regulators of apoptosis, cellular sphingolipids appear to play a significant role and have attracted increasing interest in relation to cancer. Sphingolipids are membrane and intracellular lipids that typically play structural roles and can act as signalling molecules and/or modulators of signalling pathways that are strictly connected to cell survival. In this way, sphingolipids are key regulators of a vast number of cellular processes such as cell growth, adhesion, migration, senescence, and cell death in the form of apoptosis, autophagy, and necrosis [7–9]. The sphingolipid network is complex and constituted by various molecules that, beginning from the base molecule, ceramide (Cer), are elaborated by various enzymes, mainly by the addition of phosphocholine to give sphingomyelin (SM) or the addition of monosaccharides to give glycosphingolipids (GSLs). Sphingosine-1-phosphate (S1P) is closely interconnected with this system and its content is in dynamic balance with Cer levels (this is known as “sphingolipid rheostat”) [10]. Although S1P and ceramide mainly act as signalling molecules, complex glycosphingolipids are typically clustered in plasma membrane lipid rafts, where they interact with growth factor receptors, integrins, and key molecules such as tetraspanins and caveolin and, thus, participate in intracellular signalling and cell-cell and cell-matrix interactions [11].

Cancer cells typically show significant alterations in the profiles and content of sphingolipids and S1P, which are related to aberrant cell growth, survival, and adhesion [11]. This review focuses on the roles of sphingolipids in cancer cells and their part in apoptosis and drug resistance.

## 2. Sphingolipids: Insight into Their Structure and Metabolism

**Figure 1.**
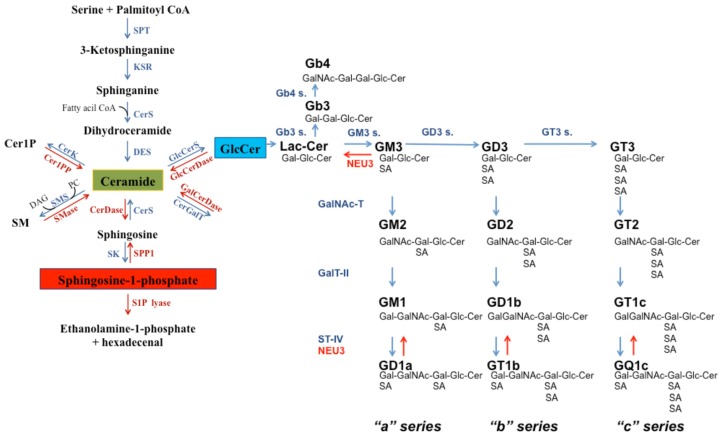
Sphingolipid synthesis and metabolism. SPT, serine-palmitoyl transferase; KSR, ketosphinganine reductase; CerS, ceramide synthase; DES, dihydroceramide desaturase; GlcCerS, glucosylceramide synthase; GlcCerDase, glucosylceramidase; CerGalT, ceramide galactosyl transferase; GalCerDase, galactosylceramidase; Cer1PP, ceramide-1-phosphate phosphatase; CerK, ceramide kinase; SMS, sphingomyelin synthase; SMase, spingomyelinase; SK, sphinosine kinase; SPP1, S1P phosphatase; Cer, ceramide; SM, Sphingomyelin; Cer1P, Ceramide-1-phosphate; GlcCer, Glucosylceramide; GalCer, Galactosylceramide; PC, phosphatidylcholine; DAG, diacylglycerol.; Gb3 s., Gb3 (globotriaosylceramide) synthase; Gb4 s., Gb4 (globotetraosylceramide) synthase; GM3 s., GM3 synthase (ST3 β-galactoside- α-2,3-sialyltransferase-5); GD3 s., GD3 synthase (ST8 α-*N*-acetyl-neuraminide-α-2-8- sialyltransferase); GT3 s., GT3 synthase (ST8 α-*N*-acetyl-neuraminide-α-2,8- sialyltransferase 1); GalNAc-T, β-1,4-*N*-acetyl-galactosaminyltransferase-1; GalT-II, galactosyltransferase; UDP-gal, βGlcNAc-β-1,3-galactosyltransferase; ST-IV, ST6 (α-*N-*acetyl- neuraminyl-2,3-β-galactosyl-1,3)-*N*-acetylgalactosaminide-α-2,6-sialyltransferase.

The different sphingolipids can originate from “*de novo* synthesis”, the degradation of complex sphingolipids [12], or the recycling of long chain bases through a salvage pathway (Figure 1) [7,13].

The “*de novo*” biosynthesis of sphingolipids begins with the condensation of palmitoyl-CoA with l-serine to form 3-ketosphinganine, which is subsequently reduced to sphinganine. Then, sphinganine binds to a fatty acid, forming dihydroceramide in a reaction that is catalysed by (dihydro)-ceramide synthase (CerS). In the salvage pathway, the same enzyme catalyses the synthesis of Cer from sphingosine (Sph) [7]. In mammalian cells, six different isoforms of CerS have recently been identified, and these are encoded by six different genes, which are members of the LASS (Longevity Assurance Genes) family. Each CerS synthesises a different Cer species, which differ from each other in fatty acid chain length and unsaturation [14,15]. In “*de novo*” biosynthesis, dihydroceramide is desaturated with the consequent formation of Cer [16]. All of the enzymes involved in the *de novo* biosynthesis of Cer are localised in the endoplasmic reticulum (ER) membrane and act on the cytosolic surface of this subcellular organelle. The products of the reactions that are catalysed by these enzymes remain anchored to the ER.

Cer is the common precursor for the synthesis of all complex sphingolipids and can be bound to different functional groups, generating Cer 1-phosphate, SM, and GSLs [7]. In particular, Cer can be phosphorylated by Cer kinase (CK) consequently forming Cer 1-phosphate (Cer1P) [7,17]. SM synthesis is based on the transfer of phosphocholine from phosphatidylcholine to Cer, with the production of a diacylglycerol (DAG) molecule and this reaction is catalysed by SM synthase (SMS). Several studies suggest the existence of two enzymatic SMS isoforms: SMS1, which is localised on the luminal side of the cis/medial Golgi apparatus; and SMS2, which is primarily localised at the plasma membrane [7,18]. Experimental evidence has shown that approximately 90% of the *de novo* synthesis of SM occurs in the cis/medial Golgi, and only a small percentage occurs at the plasma membrane. Therefore, most SM biosynthesis requires a mechanism of Cer transport from the ER, where it is synthesised, to the Golgi apparatus [19]. The localisation of SMS2 at the plasma membrane suggests its role as a regulator of SM and Cer levels for signalling pathways and signal transduction.

Glycosphingolipids are derived from the conjugation of a primary alcoholic residue of Cer with one or more saccharide units through a β-glycosidic bond. Ceramide galactosyl transferase synthesises galactosylceramide from Cer and UDP-galactose [12] whereas glucosylceramide is obtained from Cer and UDP-glucose through a reaction that is catalysed by GlcCer synthase (GCS), which is localised to the cytosolic leaflet of the Golgi apparatus [20]. Therefore, GlcCer biosynthesis, as well as that of SM, requires an efficient transport mechanism for Cer from the cytoplasmic side of the ER to the cytoplasmic side of the *cis*-Golgi [19]. Once synthesised, GlcCer can be routed directly to the plasma membrane (via a vesicular system), or it can be further modified by subsequent glycosylations, with the consequent production of more complex glycosphingolipids, such as gangliosides. This process is catalysed by different glycosyl-transferases that act by associating with individual GlcCer saccharide units, in a precise, sequential order. The enzymes involved in these reactions are located and act at the luminal surface of the Golgi cisternae and a protein that mediates the translocation of GlcCer from the cytosolic to the luminal side of the *cis*-Golgi so that subsequent glycosylations can take place, has been hypothesized [21,22]. Lactosylceramide is the basis of all complex glycosphigolpids because it is the acceptor for various transferases that generate the three major classes of complex glycosphingolipids: the lacto(neo) series (major core structure, Galβ 4GalNAcβ 3Galβ 4Glcβ Cer), the globo series (major core structure, Galα 4Galβ 4Glcβ Cer), and gangliosides (major core structure, Galβ 3GalNAcβ 4Galβ 4Glcβ Cer) [23]. In particular, gangliosides are characterised by the presence of one or more sialic acid residues in their carbohydrate moiety and are synthesised via three major pathways named “a” (GM2, GM1, and GD1a), “b” (GD3, GD2, GD1b, and GT1b), and “c” (GT3, GT2, GT1c, and GQ1c), which have a common precursor (GM3).

Membrane glycosphingolipids are constitutively degraded by a process involving endocytosis and the endo-lysosomal district. The enzymatic steps of this degradation process include exoglycohydrolases that require an acidic pH, which is guaranteed within lysosomal or endosomal vesicles, to perform their catalytic activity. These enzymes catalyse glycosphingolipid degradation into monosaccharides and Cer components, and determine the sequential, hydrolytic detachment of monosaccharides [24]. Additionally, the catabolism of gangliosides occurs at the plasma membrane through the action of the membrane sialidase NEU3 [25], in intracellular organelles (such as nuclei, through the sialidases NEU1 and NEU3 [26], mitochondria, through the long form of the sialidase NEU4 (NEU4L) [27]), and in the cytosol through the sialidase NEU2 [28].

SM degradation is catalysed by sphingomyelinase (SMase), which is an enzyme that can hydrolyse the SM phosphodiester bond, with the consequent formation of Cer and phosphocholine. Three major groups of this enzyme have been described and are distinguished according to their subcellular localisation and optimum pH: acidic, neutral, and alkaline SMases. The acidic SMase is mainly located in the lysosomal compartment. Another isoform of acidic SMase that is a splice variant of the encoding gene has been identified; this enzyme can be secreted or localised in the outer membrane leaflet [7,29,30]. The neutral SMases exist as a number of closely related isoforms that have various subcellular locations, including the inner leaflet of the plasma membrane, ER, Golgi, and even the nucleus [29,31]. The localisation of the alkaline SMase is more restricted, being mainly expressed in the intestinal tract and bile, where it participates in SM digestion [32].

Cer is degraded by ceramidases (CDases), and three isoforms of CDases have been identified and classified based on their optimal pH: acidic, neutral, and alkaline. These CDases are located in the plasma membrane, lysosome, and ER/Golgi complex, respectively [33–37]. These enzymes hydrolytically cleave Cer into fatty acids and Sph. Notably, the origin of Sph is exclusively catabolic because it is only derived from sphingolipid degradation [38].

Cer-derived Sph can be recycled or undergo phosphorylation at position C1 with the generation of sphingosine-1-phosphate (S1P) by sphingosine kinases. The existence of two isoenzymes, sphingosine kinase 1 (SK1) [39] and sphingosine kinase 2 (SK2) have been demonstrated [40,41]. S1P can be metabolised through irreversible cleavage to hexadecenal and phosphoethanolamine in a reaction that is catalysed by the S1P lyase enzyme, which is located on the cytosolic side of the ER [42]. S1P can also be dephosphorylated back to Sph through a reaction that is catalysed by lipid phosphatase or S1P specific phosphatases [43–45]. An overview of the sphingolipid biosynthetic and catabolic pathways is shown in Figure 1.

## 3. Apoptosis Induction by Chemotherapeutic Drugs

**Figure 2.**
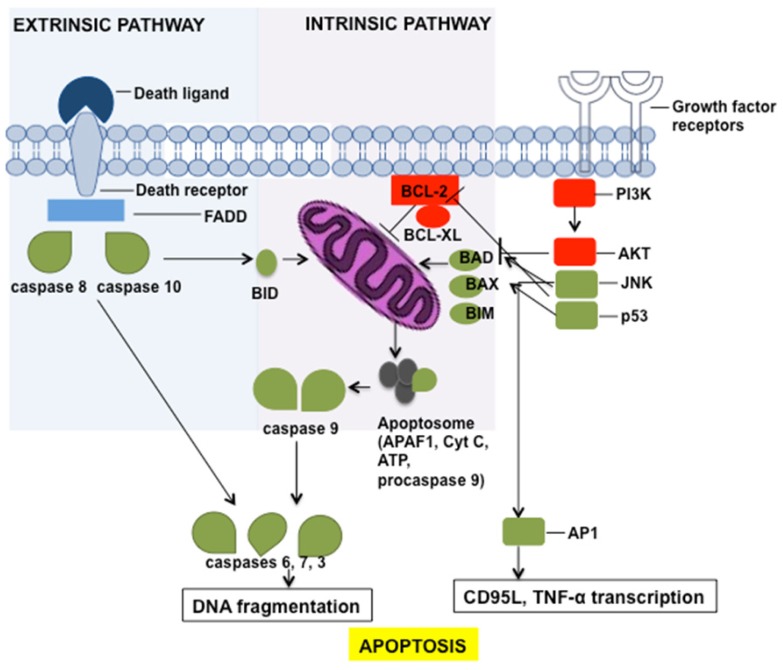
Apoptosis pathway. Schematic picture of extrinsic and intrinsic pathways of apoptosis and their main regulators. Pro-apoptotic regulators are indicated in green; anti-apoptotic regulators are indicated in red.

Two alternative pathways control apoptosis: one is mediated by death receptors that are exposed on the plasma membrane (the extrinsic pathway), and the other is mediated by the involvement of mitochondria (the intrinsic pathway) [46,47]. The final steps of both pathways involve cysteine aspartyl-specific proteases (caspases) and lead to apoptotic cell death (Figure 2).

Death receptors involved in the “extrinsic pathway” are members of the tumour-necrosis factor (TNF) receptor superfamily, and include CD95, TNF-related apoptosis-inducing ligand-R1 (TRAIL-R1) and TNF-related apoptosis-inducing ligand-R2 (TRAIL-R2). These proteins are characterised by an intracellular domain that is referred to as the “death domain” [48,49]. Decoy receptors, by contrast, are a non-signalling subset of the TNF receptor superfamily and are closely related to death receptors; however, they lack a functional death domain and have attenuated death receptor function [50].

Death receptors are activated by TNF superfamily members and recruit the intracellular FAS-associated death domain protein (FADD) through their death domains, which, in turn, attracts and activates caspases 8 and 10 [51,52]. In some cells, this pathway is insufficient to trigger apoptosis, and the involvement of mitochondria is required (intrinsic pathway). To this end, the BCL-2 family protein BID is cleaved by caspases 8 and 10 and moves to the mitochondria. The dissipation of the mitochondrial membrane potential precedes the release of cytochrome c and other apoptogenic molecules. In the cytosol, cytochrome c forms a complex with the apoptotic inactive initiator caspase activating factor-1 (APAF1), ATP, and the inactive procaspase-9. Within this complex, termed the “apoptosome”, caspase 9 is activated [53]. The activation of caspases proceeds along a cascade that ultimately leads to the activation of caspases 3, 6, and 7 by proteolysis [54]. The effector caspases cleave each other, which amplifies the process, nuclear laminin which is involved in chromatin condensation, and the inhibitor of the DNA fragmentation factor (ICAD) which leads to the release of endonucleases that fragment the DNA. Moreover, caspases cleave cytoskeletal proteins, including actin, plectin, Rho kinase 1, and gelsolin [55].

Cell cycle progression and cell death are intimately connected through p53. The rapid induction of p53 function is achieved through post-translational mechanisms, including phosphorylation and acetylation. p53, in turn, induces the expression of proteins involved in the mitochondrial pathway, such as BAX, NOXA, PUMA, and p53AIP1, and in the death receptor pathway, such as CD95, TRAIL-R1, and TRAIL-R2 [56–59].

Many drugs that have been adopted for cancer therapy induce apoptosis by differentially modulating the above-described pathways. Traditional anti-cancer drugs are classified as DNA-damaging agents, anti-metabolites, mitotic inhibitors, nucleotide analogues, or topoisomerase inhibitors [60]. By contrast, targeted drugs act by blocking specific survival signalling pathways that are typically triggered by kinases or growth factor receptors [61] and induce cell death by a mechanism known as “oncogenic shock”, which is an imbalance in pro-survival and pro-apoptotic signals that is caused by the inhibition of oncogenic kinases [62].

The survival signals that are transmitted by growth factors, cytokines, and adhesion molecules are mediated by the phosphatidylinositol 3-kinase (PI3K)/AKT pathway [63]. Anoikis is a particular type of apoptosis that is triggered by the lack of cell-matrix contacts; in fact, integrin activation usually leads to the activation of the PI3K/AKT cascade [64]. Therefore, targeted drugs also stimulate apoptosis by down-regulating PI3K/AKT (Figure 2).

Another pathway that can be activated in response to chemotherapy is the stress-activated protein kinase (SAPK, also known as JUN-*N*-terminal kinase or JNK) pathway. SAPK can regulate the activity of AP-1 transcription factors, and known pro-apoptotic target genes for AP-1 are Fas and TNF-α [65] (Figure 2).

Many drugs directly trigger the mitochondrial apoptotic pathway by down-regulating the expression of anti-apoptotic proteins belonging to the BCL-2 family of genes, mainly BCL-2, BCL-XL, and MCL1. BCL-2 family members are transcriptional targets of pro-survival transcription factors such as nuclear factor-κB (NF-κB) and signal transducer and activator of transcription 3 (STAT3) and can, therefore, be activated by oncogenic mutations in kinases [61].

## 4. The Role of Sphingolipids in Apoptosis and Apoptosis Resistance

### 4.1. Ceramide

Cer is known to play an important role in the regulation of cell fate by being directly involved in the regulation of mechanisms that control growth arrest, differentiation, and senescence [66,67]. Cer has emerged as a critical mediator of cell death. An increasing amount of evidence demonstrates that Cer also plays an important role as a tumour suppressor lipid because it is able to induce anti-proliferative and apoptotic responses; therefore, it acts as a major player in the mechanism of action of many chemotherapeutic drugs [68–71].

The molecular pathways and regulators involved in the effects of many cancer drugs include Cer metabolism. Many anticancer drugs, such as daunorubicin [72], cannabinoids [73], and etoposide [74], and other stress-causing agonists, such as Fas ligands [75], cause an increase in endogenous Cer levels through *de novo* synthesis. Furthermore, TNF-α in breast cancer cells [76] and ethanol in hepatoma cells [77] increase endogenous Cer levels, which induces SM hydrolysis by SMases. Interestingly, recent findings demonstrated that the catabolism of GlcCer into Cer by the nonlysosomal β-glucosidase GBA2 occurs at the ER and promotes apoptosis through ER stress in melanoma cells [78], thus involving glycolipid catabolism as a mechanism for the generation of proapoptotic ceramide at the ER and consequent cell death. By contrast, the overexpression of GCS, which catalyses the biosynthesis of GlcCer, decreases the endogenous Cer levels, resulting in the development of a multidrug resistance phenotype in many cancer cells [79]. Moreover, up-regulation of GCS, and, consequently, of GlcCer levels, has been found in different drug-resistant cancer cell lines and in tumor samples from patients exhibiting chemotherapy resistance, whereas GCS inhibitors can induce the death of multi-drug resistant (MDR) cells and augment the proapoptotic activity of different anticancer agents [69,71,80–82]. Furthermore the enrichment of galactosylceramide, which is synthesised from ceramide and UDP-galactose, favours leukaemia cell survival inhibiting drug-induced apoptosis [83]. Owczarek and collaborators demonstrated that high expression of ceramide galactosyl transferase accompanied by the accumulation of galactosylceramide in breast cancer cells increased tumour cell resistance to apoptosis, which suggests that the accumulation of galactosylceramide in tumour cells inhibits apoptosis [84]. In addition, in leukaemia cells, the activation of sphingomyelin synthases, which are responsible for the convertion of ceramide to sphingomyelin, is associated with the chemoresistant condition [85] and resistance to stress-induced apoptosis [86–88]. These data suggest that ceramide-based strategies promise to be effective in enhancing drug cytotoxicity and overcoming drug resistance [69,80–82]. On the basis of these findings, Cer metabolism has been identified as a feature of many drug-resistant cancers [82], and the elevation of ceramide levels through exogenous delivery, stimulation of *de novo* synthesis, or inhibition of ceramide metabolism/utilisation for the biosynthesis of complex sphingolipids has become an attractive chemotherapeutic strategy [89]. Moreover, Cer is transported from the ER to the Golgi via vesicular or monomeric transport by the ceramide transfer protein (CERT), and the latter specifically links Cer to the synthesis of sphingomyelin [90]. Two papers indicate that CERT is involved in chemoresistance and in inhibiting autophagic cell death and apoptosis in response to paclitaxel [91,92]. The expression of CERT, whose down-regulation sensitises cancer cells to multiple cytotoxic agents, is increased in drug-resistant cell lines and in residual tumour following paclitaxel treatment of ovarian cancer, suggesting that CERT could be a target for chemotherapy-resistant cancers [91]. Furthermore, Swanton and collaborators found that in HER2+ breast cancer, CERT protein expression acts as an independent prognostic variable and predictor of outcome in adjuvant chemotherapy-treated patients [92]. By contrast another paper implicated the loss of CERT in the progression of triple-negative breast cancer cells, which express cytokeratins 5/6, 14, and 17, lack ER/PR expression and ErbB2/HER2 amplification/overexpression and are especially refractory to treatment [93], thus suggesting that the role of CERT in chemoresistance and cancer progression is still controversial and could strongly depend on the global cancer gene expression profile.

As in other tumour cells, an increase in Cer is important for the activity of many cytotoxic treatments in glioma cells [94–96]. In particular, the accumulation of *de novo* synthesised Cer is crucial for cannabinoid-triggered ER stress and apoptosis in these cells [73,97], and the accumulation of Cer in the ER owing to impaired Cer traffic is associated with the antiproliferative effect of nitric oxide [98], which suggests that Cer levels in the ER can be crucial for glioma cell fate. The control of Cer levels in the ER can involve specific enzymes that utilise Cer, such as SMS and GCS [99,100], as well as the vesicular- and protein-mediated transport of Cer, from the ER to the Golgi apparatus in the sphingolipid biosynthetic pathway [19,98,101–103]. It should be hypothesised that the dysregulation of one or more of these metabolic events could be involved in the intrinsic or acquired drug resistance of glioma cells.

In support of this hypothesis, PI3K/AKT signalling, whose aberrant activation has been identified as crucial to the malignant features of glioblastomas, such as rapid tumour growth, invasiveness, and resistance to cytotoxic treatments [104], regulates sphingolipid metabolism, which promotes Cer vesicular transport and results in a reduction in ER Cer levels and increased synthesis of complex sphingolipids in glioma cells [105]. In this context, it is interesting to note that apoptotic and non-apoptotic Cer-induced cell death can be inhibited by activation of the AKT pathway. In addition, up regulation of GCS protects glioblastoma cells against autophagic and apoptotic death and contributes to cell survival under chemotherapy [81].

**Figure 3.**
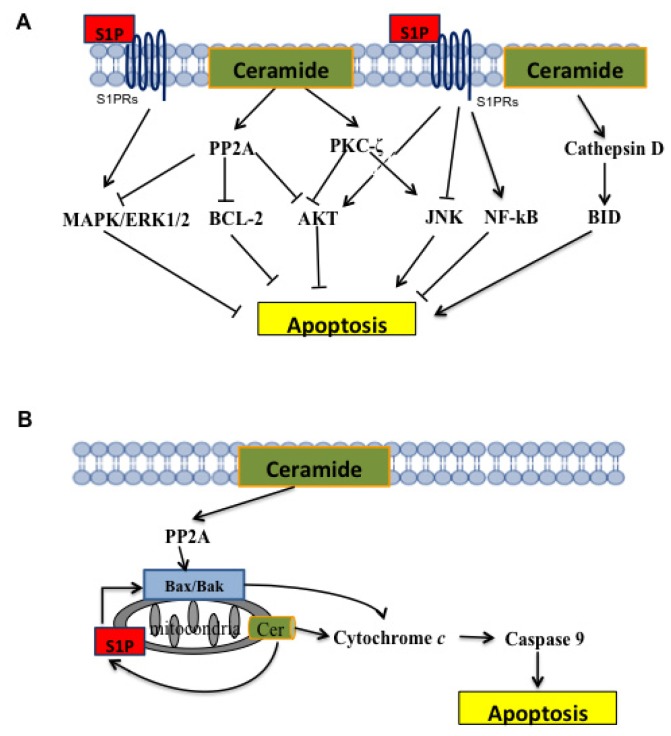
Regulation of apoptosis by sphingolipids. (**A**) Cer directly binds and activates the lysosomal protease cathepsin D to enhance BID cleavage and induction of the mitochondrial pathway of apoptosis. Cer activates PP1 and PP2A to regulate the alternative splicing of apoptosis-promoting variants BCL-xS and Caspase-9 and inhibit the antiapoptotic effects of BCL-2, respectively. Kinase signaling. Cer directly activates PKC-ζ, which mediates the activation of JNK and inhibition of AKT to promote apoptosis. S1P suppresses Cer-mediated activation of JNK and activates pro-survival Akt/mTORC1, MAPK/ERK, and NF-κB signalling pathways through cell surface receptors; (**B**) Cer assembles channels in the outer membrane of mitochondria to promote the release of cytochrome c (cyt c) for caspase-9 activation. Cer promotes BAX activation and recruitment to the mitochondria through the PP2A-dependent dephosphorylation of BAX. Furthermore, mitochondrial Cer is metabolized to S1P which directly activates BAX.

The diversity of Cer species that exist within the cell could explain the variety in Cer signalling. Cer is a substrate or product of more than 28 distinct enzymes and consists of more than 200 individual species with varying acyl chain lengths [106]. Hannun and Obeid hypothesised that individual Cer molecules are generated within distinct biochemical pathways and subcellular compartments to exert unique functions [106]. Further investigation and MS/MS lipidomics will contribute to identifying individual Cer species and their respective biological effects. Cer is an intracellular messenger that is able to regulate many intracellular effectors that mediate activation of the apoptotic process. In particular, Cer can activate serine/threonine protein phosphatases (PP1 and PP2A). These phosphatases act on several substrates that are all implicated in cell pathways that regulate proliferation and apoptosis. Among the substrates for PP1 and PP2A are retinoblastoma protein (pRB), BCL-2, c-JUN, SR proteins, and AKT (Figure 3A). Cer-mediated activation of PP1 seems to be involved in the cell cycle arrest in the G1 phase that is due to dephosphorylation of pRB [68,107]. Furthermore, PP1 induces the dephosphorylation of SR proteins, a family of serine/arginine-domain proteins that are known modulators of mRNA splicing, thus inducing the alternative splicing of genes encoding BCL-X and caspase-9 to generate pro-apoptotic splice variants [75,108]. The mitochondrial membrane potential can also be altered by Cer, most likely through PP2A-mediated dephosphorylation of BCL-2, which cause its inactivation and favours the apoptotic process [109]. Cer also activates Cathepsin D protease, which, in turn, recruits and activates the pro-apoptotic protein BID to induce apoptosis (Figure 3A) [68]. PKC-ζ is another important target that is phosphorylated and, thus, activated by Cer. Activated PKC-ζ, in turn, mediates the activation of JNK and inhibition of AKT to promote apoptosis (Figure 3A) [110].

Cer can also exert an antiproliferative role through inhibition of the mitogen activated protein kinase (MAPK) pathway, which promotes the dephosphorylation of the serine/threonine kinases extracellular signal regulated protein kinase 1 and 2 (ERK 1/2) (Figure 3A) [111]. These proteins, when dephosphorylated, are inactive and not able to migrate into the nucleus to promote the expression of genes involved in cell proliferation [112–116].

Cer can self-associate within the plane of the membrane bilayer and then fuse with GSL- and cholesterol-containing rafts, resulting in the formation of signalling platforms [117]. Some stimuli that activate acid SMase at the plasma membrane induce the formation of ceramide-enriched domains that trap and cluster signalling proteins [117], and some proteins associated with the apoptotic signalling Fas receptor FADD and caspase 8 have been shown to cluster within ceramide-enriched domains [118,119].

Studies over the past two decades have implicated the mitochondria as a key site of ceramide-mediated apoptosis [110]. Early investigations have demonstrated direct effects of Cer on mitochondrial function.

Mitochondrial Cer levels are increased during apoptosis in response to diverse stimuli, including CD95/Fas and radiation [120]. Furthermore, selective targeting of bacterial SMase to various organelles has revealed that a mitochondrial pool of Cer is sufficient to induce apoptosis in MCF7 breast adenocarcinoma cells [121]. Furthermore, Cer can induce apoptosis by forming membrane channels in mitochondria that are large enough to release cytochrome c [122]. In addition, Cer has been reported to induce BAX dependent apoptosis in several cancers (Figure 3B) [110], including glioblastoma [123], breast cancer [124], prostate cancer [125], colon cancer [125], and acute myeloid leukaemia [126].

Chipuk and coworkers demonstrated that Cer transfer to the mitochondria is functional to form S1P and/or hexadecenal, which directly promote BAX and BAK activation, respectively [127]. Therefore, local regulation of sphingolipid metabolism within mitochondria appears to play a critical role in apoptosis (Figure 3B) [128]. In addition to the potential direct lipid effects, C2-ceramide enhances the activation and translocation of BAX through the dephosphorylation of Ser184, which occurs through PP2A [129]. In particular, PP2A dephosphorylates BAX at Ser184 *in vitro* and interacts with BAX upon Cer treatment in intact cells [129].

### 4.2. Sphingosine-1-Phosphate

**Figure 4.**
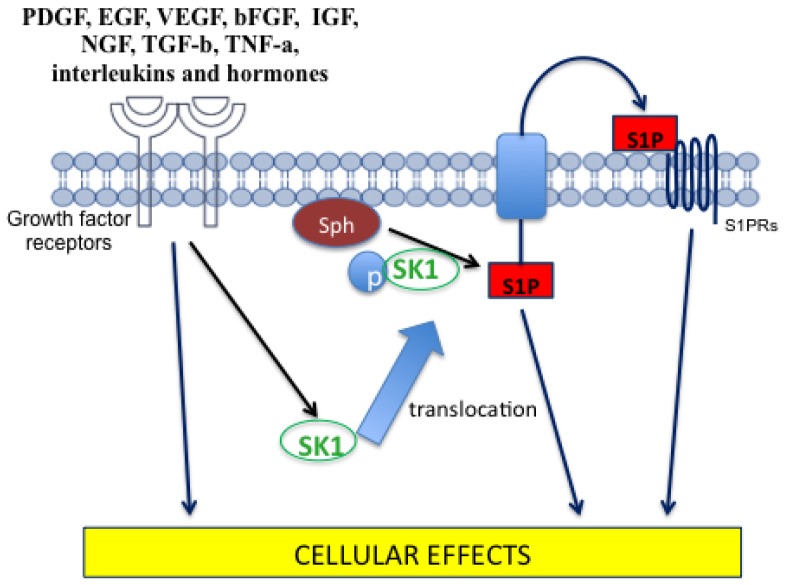
SK1 activation by various agonists (PDGF, EGF, VEGF, bFGF, IGF, NGF, TGF-β, TNF-α, interleukins and hormones) via their receptor is followed by SK1 translocation to the plasma membrane to generate S1P from sphingosine.

S1P is a bioactive molecule that has antiapoptotic properties through antagonising ceramide (Cer)-mediated apoptosis by activating ERK and suppression of ceramide-induced JNK activation [10]. In particular, growth factors such as PDGF, EGF, VEGF, bFGF, IGF-1, nerve growth factor (NGF), and TGF-β), cytokines (such as TNF-α, interleukins), and hormones stimulate SK1 activation [130] by inducing its phosphorylation on Ser225 in a PKC- and ERK-dependent manner (Figure 4). This modification leads to SK1 translocation from the cytosolic compartment to the plasma membrane. Because sphingosine is mainly in the inner layer of the plasma membrane, SK1 translocation to the plasma membrane is essential for sphingosine phosphorylation and the consequent generation of S1P (Figure 4) [131].

Further characterisation of S1P-mediated cellular effects has revealed that the SK1 and SK2 isoforms differentially regulate cell fate [110], and fundamental roles of S1P and SK1 in favouring cell survival, migration, angiogenesis, and drug resistance have been identified [68,132]. A significant body of literature implicates SK1/S1P signalling in the process of drug resistance because this signalling protects cancer cells from chemotherapy-induced apoptosis. For example, in prostate adenocarcinoma SK1 regulates drug-induced apoptosis and serves as a chemotherapy sensor in culture and animal models. In particular, SK1 overexpression has been show to impair the efficacy of chemotherapy (docetaxel and camptothecin), and the silencing of SK1 by siRNA or its pharmacologic inhibition has been show to induce apoptosis *in vitro* and *in vivo* [133]. Moreover, Bektas and coworkers demonstrated that over-expression of SK1 reduced the sensitivity of melanoma cells to Fas- and Cer-mediated apoptosis, and that this effect could be reversed by inhibiting SK1 expression [134]. Furthermore, it has been shown that SK1 over-expression increases the proliferation and resistance to tamoxifen of breast cancer cells, whereas knock down of SK1 restores tamoxifen responsiveness [135]. Interestingly, during the onset of TNF-induced apoptosis, cathepsin B is responsible for the proteolysis of SK1, which involves the “lysosomal pathway” of apoptosis in its down-regulation [136]. Despite advances in the knowledge of SK2, much is still unknown about this kinase. While SK1 activity is associated with mitogenic and anti-apoptotic effects, over-expression of SK2 has been shown to promote cell death [110,137–139]. SK2 localises primarily in the nucleus and its over-expression suppresses growth and enhances apoptosis, preceded by cytochrome c release and activation of caspase-3 [139]. Moreover, SK2 contains a BH3 domain that sequesters BCL-XL and abrogates its anti-apoptotic function indicating that SK2 can be pro-apoptotic [138]. It has been shown that SK1 is an oncogene that is up-regulated in many cancers and associated with drug resistance [140], whereas the expression of SK2 sensitises cells to chemotherapeutic agents [141]. It has been demonstrated that glioblastoma cell lines and tissue specimens show high SK1 expression [142,143], which correlates with worse prognosis and poor patient survival [140], and that silencing or pharmacological inhibition of SK1 and SK2 (i) decreases the proliferation rate of glioblastoma cells, which prevents their entry into the cell cycle [140,142]; and (ii) restores the sensitivity of glioma stem cells to temozolomide [144]. SK2, has been demonstrated to play a role in a sphingosine salvage pathway of mammalian cells that acts in concert with S1P phosphatase (SPP1) to convert S1P back to sphingosine, which, in turn, is converted to Cer for Cer production, while cytosolic SK1 generates S1P, which may decrease the generation of Cer [44,145]. Taken together, this evidence demonstrates a relationship between the changes in S1P metabolism and the development of drug resistance in human cancer cells.

S1P can exert its bioactive effects intracellularly [132] by acting as a second messenger, and in the extracellular milieu, where it mainly acts as a ligand for the specific cell surface G-protein coupled receptors [110,146–148] S1P1–S1P5, which are coupled to various signaling pathways [110], such as Akt/mTOR [149], NF-κB [150], and MAPK [151] (Figure 3A).

For example, S1P which acts intracellularly, activates calcium channels in a pertussis toxin- independent manner to mobilize calcium stores [152]. Recently Spiegel and collaborators demonstrated that SK2-mediated generation of nuclear S1P inhibits the activity of histone deacetylase 1 and 2 (HDAC1 and HDAC2) to regulate gene expression [153], identified S1P as the first nuclear lipid to be associated with the epigenetic regulation of gene expression, and exposed nuclear sphingolipid metabolism as an intriguing area of study for the regulation of autophagy and apoptosis. Furthermore as previously noted, the generation of mitochondrial S1P directly activates BAK to promote the release of cytochrome c, therefore, further stressing the importance of subcellular sphingolipid pools in regulating cell fate [127].

The use of different inhibitors of SKs highlights the key anti-apoptotic role of SKs and has proven useful in enhancing the cytotoxic effects of different chemotherapeutics. Screening a large library of non-lipid, synthetic inhibitors of SK1, French *et al*. [154] found that each inhibitor induced apoptosis concomitant with the tumour cell cytotoxicity of various cancer cell lines, including multidrug-resistant lines. The non-specific inhibitor of SKs dimethylsphingosine (DMS) has been shown to inhibit leukaemia, colon, epidermoid, and lung tumour cell growth and to reduce metastasis *in vivo* [155,156], as well as increase the sensitivity of human leukaemia cells to apoptosis in response to radiation, TNF-α and Fas ligands [157]. A DMS-mediated decrease in cell viability and invasion and increase in apoptosis may be triggered by the activation of p38 and the SAPK/JNK signaling pathways [158]. Safingol, a competitive SK1 inhibitor, has been found to induce apoptosis and increase the growth-inhibitory actions of doxorubicin, even in multidrug-resistant cancer cells and is under evaluation for the treatment of various human cancers in combination with other chemotherapeutic agents, such as cisplatin [159].

However, because SK1 and SK2 could perform different functions in cancer progression and drug resistance, it is very important to have specific inhibitors for these isoforms. SK1-I (BML-258), which is a specific SK1 inhibitor, prevents tumour growth and vascularisation, induces apoptosis in glioblastoma xenografts, and enhances survival in orthotopic glioblastoma [160]. A SK2-selective inhibitor (ABC294640) inhibits tumour growth and induces apoptosis and autophagic cell death in kidney tumour xenografts [161]. In addition to its immunosuppressive function, the S1PR antagonist FTY720 induces growth arrest and apoptosis in leukaemia, bladder, prostate, breast cancer, and glioma cells [162–165]. FTY720 also promotes apoptosis in drug resistant multiple myeloma cells, by inducing mitochondrial membrane potential changes, Bax cleavage, and caspase activation [166]. Furthermore, FTY720 is able to prevent tumour growth and metastasis in mouse breast cancer cells, *in vitro* and *in vivo* [167].

Lépine and her collaborators demonstrated that doxorubicin treatment dramatically reduces the autophagic activity of SPP1-depleted cells and sensitises the cells to apoptosis, which surprisingly, occurred in an autophagy-dependent manner [168]. The authors concluded that doxorubicin enhances *de novo* Cer synthesis to suppress Akt and stimulate the calpain-mediated cleavage of Atg5, thus effectively switching protective autophagy in SPP1-depleted cells to apoptosis [168]. Moreover, different sphingosine kinase inhibitors stimulate autophagy [161,169]; SKI-I has been demonstrated to stimulate the autophagy-dependent activation of caspase-8 and initiation of the caspase cascade [169]. In contrast, DMS, SKI-2, and ABC294640 induced the autophagic cell death that was associated with a decrease in AKT activity and upregulation of Beclin 1, similar to the mechanisms described for Cer [161,170]. The mechanisms underlying the anti-apoptotic effect of S1P appear to be primarily the prevention of cytochrome c release from mitochondria and inhibition of changes in the mitochondrial membrane potential [157]. However, further studies are required to characterize the effects of SK1 and SK2 in the regulation of cell survival or cell death.

## 5. The Role of Complex Glycosphingolipids in Apoptosis and Apoptosis Resistance

### 5.1. Gangliosides

A great number of studies have demonstrated that cancer cells endowed with apoptosis resistance also display a modified ganglioside pattern. In ovarian carcinoma cells, fenretinide and paclitaxel resistance have been correlated with alterations in the cell ganglioside composition [171,172]. The connections between these modifications and the mechanisms leading to drug resistance have only been clarified in part, and it is possible that multiplex mechanisms connect ganglioside alterations to drug resistance. In some drug-resistant cancer cells, an increase in ganglioside synthesis appeared to be a tool for escaping the accumulation of Cer [171,173], which, as previously described, is a key mediator of apoptosis induced by many anti-tumour drugs. In fact, in this way, Cer is converted into GlcCer and complex glycosphingolipids, including gangliosides, which are not endowed with the same pro-apoptotic capability. In other reports, it has been shown that gangliosides are directly connected to the expression and activation of P glycoprotein (P-gp) and the multidrug resistance-associated protein (MRP1) [174,175]. In many cases, cellular ganglioside profiles are connected with cell differentiation [176–178]; therefore, ganglioside modifications that are able to induce a more differentiated phenotype are also frequently associated with a decrease in apoptosis resistance [179]. Along these lines, an increase in GM3 was demonstrated to promote the differentiation of human colonic carcinoma cells [180] and the chronic myeloid leukaemic cells, K562 [181]. In both of these cases, this event also induced increased apoptosis sensitivity. Gangliosides are also implicated in the regulation of the epithelial mesenchymal transition (EMT) [182], which is the conversion from a less mobile epithelial phenotype to a mesenchymal phenotype with higher mobility and resistance to apoptosis, particularly anoikosis [183].

The altered ganglioside pattern could also reflect the composition of membrane lipid rafts and, therefore, the activation of receptors and signalling molecules that are clustered in these regions of the plasma membrane and are involved in the control of apoptosis.

Some recent papers demonstrated that the treatment of cancer cells with the TNFα-related apoptosis inducing ligand (TRAIL) induces the redistribution of TRAIL death receptor 1 (DR4) and TRAIL death receptor 2 (DR5) into lipid rafts and, thus, triggers apoptosis through the activation of caspases 8 and 10 [184–186]. Moreover, the recruitment or constitutive localisation of DR4 and/or DR5 into lipid rafts accounts for the sensitivity of non-small cell lung carcinoma cells to TRAIL [187] and, for ursodeoxycholic acid and fludarabine-induced apoptosis in gastric cancer [186] and Burkitt lymphoma B cells [188]. The cell ganglioside composition reportedly affects the localisation of DR4 and DR5 into lipid rafts in B lymphoblastoid human cells [189]; therefore, it could be hypothesised that modifications concerning ganglioside metabolism could induce the exclusion of DR4 and DR5 from lipid rafts and, thus, cause the highly variable response of cancer cells to apoptosis during TRAIL treatments. Ganglioside alterations achieved by cancer cells could also modulate the dynamics of the plasma membrane in modifying the internalisation and recycling of key signalling molecules and receptors that are involved in the regulation of apoptosis. In renal carcinoma cells, the enrichment of ganglio-series gangliosides, in particular GD1a (αNeu5Ac(2-3)βGal(1-3)βGalNAc(1-4)αNeuu5Ac (2-3)βGal(1-4)βGlc(1-1)Cer), to the detriment of globo-series gangliosides, changes the functionality of plasma membrane caveolae and increases the endocytosis and lysosomal degradation of β1 integrin and epidermal growth factor receptor (EGFR) [190]. This event radically changes intracellular signalling, down-regulates FAK and PI3/AKT pathways, and promotes a shift from autophagy to apoptosis, which increases the occurrence of cell death after etoposide treatment [190].

Although it may be suspected that, in many cases, the overall cell ganglioside profile and the relative proportions of gangliosides are important in defining drug-resistant behaviour, the direct involvement in apoptosis control and drug resistance has been better characterised for some gangliosides than for others.

**Figure 5.**
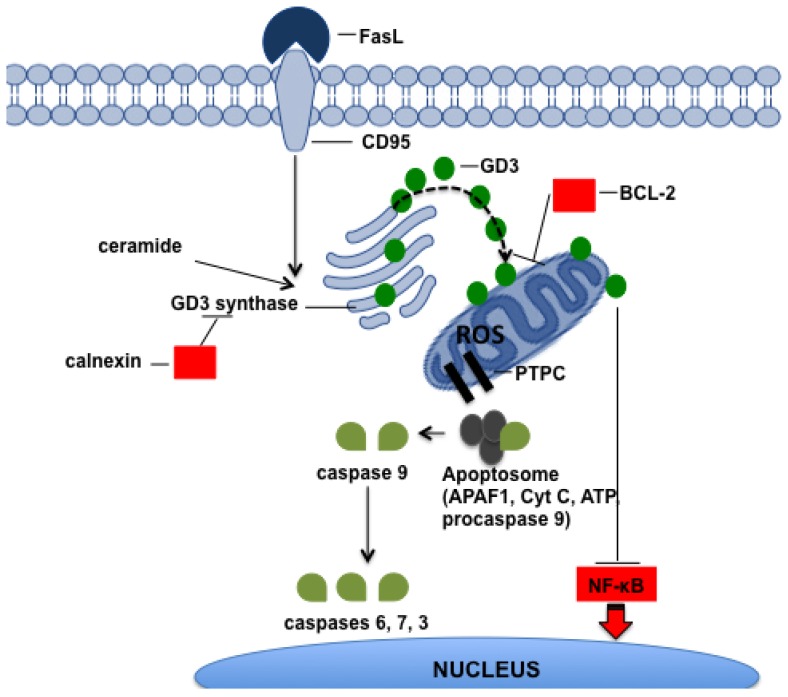
Ganglioside GD3 role in apoptosis. Involvement of ganglioside GD3 in the apoptotic cascade.

GD3 is a minor ganglioside in most normal tissues except placenta and thymus [191]. Nevertheless, GD3 is highly synthesised during development and in a variety of diseases, including many tumours, particularly, of neuroectodermal origin such as melanoma, medulloblastoma, and neuroblastoma [192] and many others such as meningioma, glioma, sarcoma, leukaemia, colorectal and pancreatic carcinoma [193,194]. In addition to its involvement in cell growth and differentiation, GD3 plays a well-recognised role in apoptosis. In human tumour lymphoid and myeloid cell lines, GD3 rapidly accumulates upon Fas triggering or Cer exposure and directly induces apoptosis (Figure 5). Preventing endogenous GD3 accumulation, by suppressing GD3 synthase expression with specific antisense oligodeoxynucleotides, can substantially block Fas- and ceramide-induced apoptosis [195]. GD3 is synthetised by a sialyltransferase (ST8 α-*N*-acetyl-neuraminide-α-2-8-sialyltransferase), which is referred to as GD3 synthase and is localised in the early Golgi. During apoptosis, GD3 relocates to the mitochondria, where it elicits a burst of ROS production from complex III of the mitochondrial electron transport chain. In this way, GD3 contributes to the opening of the mitochondrial permeability transition pore complex (PTPC) and induces the dissipation of the inner mitochondrial trans-membrane potential (DWM) [196]. These events are then associated with mitochondrial swelling, outer membrane disruption, and the release of cytochrome c, which, in turn, activates caspase-9 and, subsequently, the entire apoptotic cascade (Figure 5). PTPC opening is also controlled by BCL-2 [197] and the over-expression of BCL-2 prevents apoptosis induced by exogenous GD3 or endogenous GD3 synthesised by GD3 synthase (Figure 5) [198]. GD3 accumulation and translocation to the mitochondria requires Cer produced by acidic SMase during Fas [199] or TNFα-induced apoptosis [200]. GD3 could also play a pro-apoptotic role by preventing the nuclear translocation of NF-κB and NF-kB-dependent gene expression. In this way, GD3 sensitises cells to apoptotic stimuli [200]. These effects appear to be specific to GD3 and possibly due to the chemical features of its two sialic acid residues, because other gangliosides, such as GM3 and GD1a, are not able to dissipate the DWM [195]. Interestingly, chemotherapy and radiotherapy were demonstrated to induce the increase of GD3 in cancer cells. Fenretinide induces the activation of acidic SMase and GD3 synthase, leading to the apoptosis of neuroblastoma cells [201] and daunorubicin treatment triggers the accumulation of GD3 in human leukaemic cells [83].

Despite the numerous studies that have shown the pro-apoptotic role of GD3, the reality is that, in many cancer cells, GD3 accumulation is not lethal. In addition to its pro-apoptotic role, GD3 has been shown to promote tumorigenesis by enhancing migration, adhesion, proliferation, metastasis, and angiogenesis [202–205]. Therefore, many cancer cells find a way to accumulate GD3 and benefit from it by simultaneously defending themselves from GD3-mediated mitochondrial damage. The main tactic that cancer cells use to suppress the pro-apoptotic drive of GD3 is to modify the GD3 structure through the addition of *O*-acetyl esters to the hydroxyl group at the C9 position of sialic acid. 9-*O*-acetylation abrogates the pro-apoptotic activity of GD3 and has been recorded in many cancers including melanoma, leukaemia, and glioma [206–210]. Another strategy for suppressing the pro-apoptotic action of GD3 is to prevent its translocation to the mitochondria. When GD3 synthase binds to the molecular chaperone calnexin, this enzyme is retained within the ER and does not move to the Golgi (Figure 5). This event does not compromise the enzymatic activity of GD3 synthase, but *de novo* synthesised GD3 fails to reach the mitochondria [211]. Calnexin expression can be induced by growth factors and is up-regulated in breast cancer and myeloma [212]. BCL-2 over-expression is also useful for avoiding apoptosis in the presence of GD3 [212].

**Figure 6.**
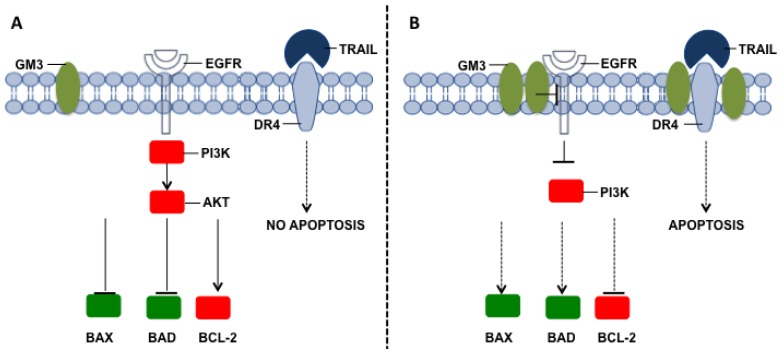
Ganglioside GM3 role in apoptosis. (**A**) Activation of pro-survival intracellular signaling pathways in cells showing a low content of GM3 in the plasma membrane; (**B**) Inhibition of pro-survival intracellular signaling pathways and activation of pro-apoptotic pathways and regulators in cells showing a high content of GM3 in the plasma membrane.

Another ganglioside that has mainly been studied in the context of apoptosis and drug resistance is GM3; however, the obtained results are controversial. In fact, an increase in GM3 in murine bladder cancer cells stimulates apoptosis [213], and in K562 human myeloid leukemic cells, it restores the sensitivity to etoposide and staurosporine treatments, which promote increased expression of the pro-apoptotic proteins BAX and BAD and decreased expression of BCL-2 [181] (Figure 6A,B). In non-small cell lung adenocarcinoma cells, the *IC*_50_ values for the EGFR receptor tyrosine kinase (EGFR-TK) inhibitors, gefitinib and AG1478, are inversely correlated with increased GM3 synthase levels [214]. Moreover, in human leukaemic B-cells a molecular interaction between GM3 and the TRAIL death receptor DR4 within membrane lipid rafts has been detected and this has been strictly related to TRAIL susceptibility of cancer cells (Figure 6A,B) [189].

By contrast, it has also been reported that the doxorubicin-resistant small-cell lung cancer cell line SBC-3/ADM100 and the cisplatin-resistant small-cell lung cancer cell line SBC-3/CDDP exhibit higher levels of GM3 than the parent cell line [215]. Similarly, transfection of the GM3 synthase (SAT-I) cDNA into J5 cells, which are GM3-deficient clones of the murine 3LL Lewis lung carcinoma cell line [216], gives rise to enhanced resistance to etoposide and doxorubicin-induced apoptosis mainly owing to the over-expression of BCL-2 [217]. To evaluate GM3 in apoptosis resistance, the chemical structure of this ganglioside needs to be more accurately determined. In fact, there is emerging evidence that cancer cells could also display variants of the common *N*-acetyl GM3, such as de-*N*-acetyl GM3, which has a free amino group at the 5 position of sialic acid instead of the acetyl group [218] or *N*-glycolyl GM3, which is a variant of GM3 that contains *N*-glycolylneuraminic acid instead of *N*-acetylneuraminic acid (Figure 6) [219–221].

These chemical modifications strongly alter the capability of GM3 to modulate the activity of EGFR: thus, *N*-acetyl GM3 inhibits EGFR autophosphorylation/activation [222], whereas *N*-glycolyl GM3 is not able to act in this manner [223], and de-*N*-acetyl GM3 even stimulates EGFR activation [224]. Because EGFR activation mediates resistance to chemotherapy and apoptosis by activating the mitogen activated protein kinases (MAPK) and phosphoinositide 3-kinase (PI3-K)/AKT-dependent survival pathways [225], the presence of different species of GM3 in tumours could explain the controversial role of this ganglioside in apoptosis, as the experimental data have shown.

### 5.2. Globosides

**Figure 7.**
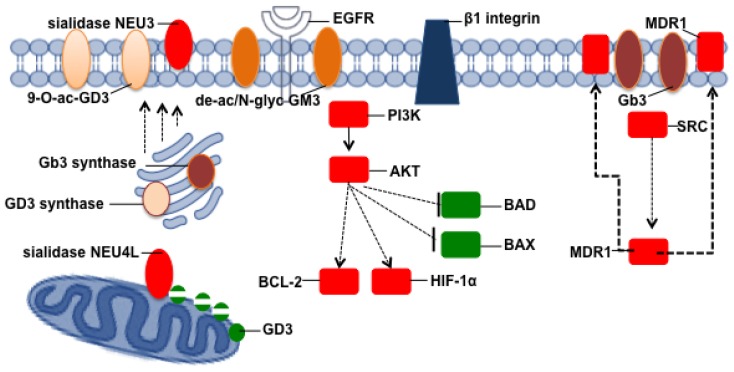
Involvement of gangliosides, globosides, and enzymes involved in their metabolism in resistance to apoptosis. Schematic picture of how particular cell glycosphingolipid profiles could lead to apoptosis resistance in cancer cells.

In addition to gangliosides, it has been demonstrated that also some globo-series glycosphingolipids are important in apoptosis and drug resistance in cancer. The globoside Gb3 (Galα 4Galβ 4Glcβ Cer), also known as CD77, was identified as a P^k^ antigen of the P blood group system and acts as a natural receptor for bacterial toxins of the Shiga family (Stx) [226]. Gb3 is highly expressed by immature B-cells and various types of cancer, including Burkitt’s lymphoma [227,228] and breast cancer [229]. Gb3 has been shown to increase the expression of the human multidrug resistance gene (MDR1) through the recruitment of c-Src kinases [230]; therefore Gb3 could enhance apoptosis resistance simply by promoting the extrusion of drugs from cancer cells (Figure 7).

The globoside Gb4 (GalNAcβ 3Galα 4Galβ 4Glcβ Cer) is synthetised at high levels by erythrocytes and during embryogenesis [231]. It has been demonstrated that Gb4 is able to interact with EGFR in colon carcinoma cell lines and to transduce survival signals mediated by the activation of MAPK [232].

## 6. Pivotal Enzymes Involved in Glycosphingolipid Metabolism and in Apoptosis Resistance

The involvement of some glycosphingolipids in apoptosis resistance entails the association of enzymes that regulate their metabolism in cancer pathogenesis. Thus, high expression of GD3 synthase (ST8 α-*N*-acetyl-neuraminide-α-2-8-sialyltransferase) is associated with poor prognosis in breast cancer [233], whereas small interfering RNAs directed towards the GD3 synthase gene induce apoptosis in lung cancer cells [234]. Along these lines, the expression of GM3 synthase (ST3 β-galactoside-α-2,3-sialyltransferase-5) mRNA has been suggested to be a novel, useful marker with which to predict the sensitivity of non-small-cell lung cancer to EGFR-TK inhibitors [214].

The plasma membrane sialidase NEU3, which is deeply involved in ganglioside catabolism [25], has been demonstrated to play a key role in determining the survival of cancer cells [235]. NEU3 is often markedly up-regulated in human cancers and leads to apoptosis resistance, deregulation of the activation of EGFR, AKT, and Ras, and the expression of BCL-XL and BCL-2 [235]. In renal carcinoma cells, NEU3 silencing promotes a shift from autophagy, which seems to be the characteristic response to etoposide treatment, to apoptosis, thus significantly increasing cell death [190]. In fact, the progression of renal carcinoma is usually related to the de-repression of LC3B, which stimulates autophagy and plays a key role in sustaining cell viability during nutrient starvation, metabolic stress, and radio/chemotherapeutical treatments [236]. NEU3 has been demonstrated to further sustain this process by increasing BCL-2 expression and decreasing BAX expression [190]. Additionally, in chronic myeloid leukaemic K562 cells, NEU3 silencing leads to increases in pro-apoptotic molecules such as BAX and BAD and decreases in BCL-2, and thus reduces the resistance to the death signals that are conveyed by etoposide and staurosporine [181]. The sialidase NEU3 has been further implicated in the mechanisms leading to apoptosis resistance in colon cancer [237], melanoma [238], and prostate cancer [239]. Moreover, it has been shown that NEU3 stimulates the EGFR signalling pathway and, in turn, the pro-survival molecules AKT and p70S6K, which ultimately stimulate the hypoxia-inducible factor (HIF-1α) and increase cell survival under hypoxic conditions [240]. Otherwise, in acute lymphoblastic leukaemia lymphoblasts, NEU3 is down-regulated, and over-expression of the enzyme leads to apoptosis triggered by the increase in ceramide [209]. Because these data show a significant connection between NEU3 and apoptosis resistance in cancer, the sialidase NEU3 has been defined as a novel oncogene [241].

Sialidase NEU4L, which is localised in the external mitochondrial membrane [242], has been demonstrated to recognise GD3 as a substrate and be involved in apoptosis regulation [27]. In particular, the expression of NEU4L dramatically decreases prior to apoptosis stimulated by catechol metabolites in neuroblastoma cells; and, in parallel, the ganglioside GD3 shifts to the mitochondria, and cytochrome c is released into the cytosol [27]. The anti-apoptotic roles of gangliosides, globosides, and the enzymes involved in their metabolism are illustrated in Figure 7.

## 7. Conclusions

Chemotherapy has rapidly evolved in recent last years; nevertheless, the onset of resistance mechanisms often impairs its long-term efficacy. Thus, strategies to circumvent therapeutic resistance by restoring apoptotic pathways could hold promise for better clinical management of patients with cancer. It is becoming increasingly evident that sphingolipids are deeply involved in the regulation of apoptosis and the apoptosis resistance that is displayed by cancer cells. Therefore, it is important to be able to regulate sphingolipid metabolism in order to develop novel anti-cancer therapeutics or to improve the effectiveness of current treatment strategies. From this perspective, the elevation of Cer levels, which has been demonstrated to have strong pro-apoptotic effects, through exogenous delivery or stimulation of *de novo* synthesis has become an attractive chemotherapeutic strategy [89].

Cancer cells usually convert Cer into GlcCer and gangliosides to evade the pro-apoptotic function of Cer. It should be noted that cancer cells often show a ganglioside-enriched profile that has been demonstrated to be associated with an apoptosis-resistant behaviour. To this end, approaches that reduce GlcCer and ganglioside synthesis could improve the efficacy of chemotherapy. For example, PDMP (1-phenyl-2-decanoylamino-3-morpholino-1-propanol) and PPMP (1-phenyl-2- palmitoylamino-3-morpholino-1-propanol) are structural analogues of ceramide that inhibit GlcCer synthase and have been demonstrated to trigger apoptosis in several cancer cell types [243,244]. Additionally, strategies that silence the expression of GlcCer synthase reverse multidrug resistance in cancer cells [80,245].

Furthermore, cancer cells qualitatively alter the chemical structure of ganglioside-originated species, such as 9-*O*-acetylated GD3, *N*-glycolyl GM3, and de-*N*-acetyl GM3, which are usually not present in healthy tissues and are involved in apoptosis resistance, as previously discussed. To this end, approaches that target GD3 acetylation have been revealed to induce apoptosis in glioblastoma cells [208]. The control of the complex glycosphingolipid profiles of cancer cells could also be restored by genetically manipulating the enzymes involved in their metabolism, such as sialidases or synthases [241].

Another way in which cancer cells escape Cer accumulation is converting Cer into S1P, which, unlike Cer, acts as a pro-survival signal. Thus, the development of compounds that are able to modulate S1P metabolism to strongly sensitise cells to chemotherapeutic treatments for triggering tumour cell death is becoming an important approach for improving the survival rates of patients with cancer [154–156,246]. Moreover, FTY720, which is phosphorylated to form FTY720-phosphate and, in turn, is an agonist of four sphingosine-1-phosphate receptors, induces growth arrest and apoptosis in leukaemia, bladder, prostate, breast cancer, and glioma cells and prevents tumour growth and metastasis in mouse breast cancer cells *in vitro* and *in vivo* [162–165,167].

Therefore, a combined therapy employing conventional or novel targeted drugs and strategies based on chemical compounds or genetical approaches to modulate Cer, S1P, and glycosphingolipid metabolism can potentially be more beneficial than monotherapy. However, some important caveats should be underlined. In particular, one problem that needs to be overcome is the multiplicity of biological events that involved sphingolipids and the redundancy of the functions of the different sphingolipid-metabolising enzymes. For these reasons, many of the compounds that are used to modulate sphingolipid metabolism exhibit non-specific effects or are not effective at tolerable doses [154]. Thus, accumulating evidence suggests that targeting sphingolipid metabolism may be helpful in overcoming drug resistance and improving cancer therapy. However, a better understanding of the role of sphingolipids in cancer in order to allow the development of more specific drug targets and inhibitors will be required before these compounds can be reliably employed in clinical trials.
